# Emergency Heart failure Mortality Risk Grade may help to reduce heart failure admissions

**DOI:** 10.1007/s12471-022-01661-3

**Published:** 2022-03-11

**Authors:** N. E. van Hattem, S. L. M. A. Beeres, B. J. A. Mertens, M. L. Antoni, D. E. Atsma, M. J. Schalij, M. C. den Haan

**Affiliations:** 1 Department of Cardiology, Leiden University Medical Center, Leiden, The Netherlands; 2 Department of Biomedical Data Sciences, Leiden University Medical Center, Leiden, The Netherlands

**Keywords:** Heart failure, Risk assessment, Hospitalisation, Emergency department, Mortality

## Abstract

**Introduction:**

Hospital admissions for heart failure (HF) are frequent and pose a heavy burden on health care resources. Currently, the decision to hospitalise is based on clinical judgement rather than on prognostic risk stratification. The Emergency Heart failure Mortality Risk Grade (EHMRG) was recently developed to identify high-risk HF patients in the emergency department (ED).

**Objective:**

To assess the ability of the EHMRG to predict 30-day mortality in Dutch HF patients visiting the ED and to evaluate whether the EHMRG could help to reduce the number of hospital admissions for decompensated HF.

**Methods:**

Patients visiting the ED for decompensated HF were included. The decision to hospitalise or discharge was based on clinical judgement. The EHMRG was calculated retrospectively. Based on their EHMRG, patients were stratified as very low risk, low risk, intermediate risk, high risk and very high risk.

**Results:**

In 227 patients (age 73 ± 12 years, 69% male) 30-day mortality was 11%. Mortality differed significantly among the EHMRG risk groups at 7‑day (*p* = 0.012) and 30-day follow-up (*p* < 0.01). Based on clinical judgement, 76% of patients were hospitalised. If decision-making had been based on EHMRG, the hospitalisation rate could have been reduced to 66% (*p* < 0.01), particularly by reducing hospitalisations in patients at low risk of death. Mortality in discharged patients, whether the decision was based on EHMRG or clinical judgement, was 0%.

**Conclusion:**

The EHMRG accurately differentiates between high- and low-risk decompensated HF patients visiting the ED, making it a promising tool to safely reduce the number of HF admissions.

**Supplementary Information:**

The online version of this article (10.1007/s12471-022-01661-3) contains supplementary material, which is available to authorized users.

## What’s new?

- The Emergency Heart failure Mortality Risk Grade accurately differentiates between low- and high-risk Dutch decompensated heart failure patients visiting the emergency department.
- The 30-day mortality in discharged patients is 0% whether the decision is based on the EHMRG or on clinical judgement.
- Routine calculation of the EHMRG at the emergency department can help in safely and substantially reducing the number of hospital admissions for HF in the Netherlands.

## Introduction

Heart failure (HF) is a major and escalating public health problem, accounting for over 30,000 hospital admissions per year in the Netherlands [1]. These hospital admissions pose a heavy burden on health care resources [2]. Patients with decompensated HF often present to the emergency department where, in daily practice, the decision as to whether to admit a patient is based on clinical judgement rather than guided by prognostic risk quantification. Accordingly, some high-risk patients are discharged whereas some low-risk patients, who can be safely treated at home, are admitted. The admission of low-risk patients leads to inefficient use of health care resources and exposes those patients to risks related to hospitalisation.

The Emergency Heart failure Mortality Risk Grade (EHMRG) was developed to identify high-risk HF patients in the emergency department [3]. This multivariate index comprises routinely collected clinical variables and predicts mortality early after discharge from the emergency department. In 2019, a prospective external validation study revealed that the EHMRG was highly predictive of 7‑day mortality, using EHMRG7, and of 30-day mortality, using a new 30-day risk score (EHMRG30-ST), among Canadian patients with acute HF [4]. Since the EHMRG30-ST calculator is not yet available, we assessed EHMRG7 in the current study and evaluated mortality rates up to 30 days.

Until now, the added value of the EHMRG has not been studied in the Dutch HF population. We hypothesise that systematic incorporation of the EHMRG in decision-making at Dutch emergency departments may help to identify low-risk patients who can be safely discharged. Thereby, it can help to reduce the number of HF admissions and associated costs in the Netherlands. Accordingly, the aim of this study was to assess the ability of the EHMRG to predict mortality up to 30 days in Dutch decompensated HF patients visiting the emergency department. In addition, we investigated whether EHMRG-based decision-making could help to safely reduce the number of hospital admissions for decompensated HF.

## Methods

A cross-sectional study was performed among all patients who visited the emergency department of the Leiden University Medical Center in 2018. Patients with a cardiologist’s final primary diagnosis of decompensated HF (leading to the ‘diagnose behandel combinatie (DBC)’ code ‘301’ in the electronic medical record) were eligible for inclusion. If a patient had multiple emergency department visits within 2018, only the first visit was included in the study. Patients with a left ventricular assist device (*n* = 8) were excluded. In addition, in line with previous EHMRG studies [3–5] patients receiving haemodialysis (*n* = 4) or palliative care because of a non-cardiac disease (*n* = 1) and patients with a ‘do not resuscitate policy’ on arrival at the emergency department (*n* = 16) were excluded. Accordingly, the study population comprised 227 unique decompensated HF patients visiting the emergency department. In all patients, the decision to hospitalise or discharge was based on clinical judgement by the cardiologist on call. The study was conducted in accordance with the Declaration of Helsinki. The institutional ethics committee approved this retrospective evaluation of clinically acquired data. This research was performed without patient and public involvement.

### Study objectives

The primary objective of this study was to assess the ability of the EHMRG to differentiate between high and low risk for 7‑and 30-day mortality in patients visiting the emergency department for decompensated HF. The secondary objective was to evaluate whether EHMRG-based decision-making could help to safely reduce the number of hospital admissions for decompensated HF.

### Data collection

Clinical and laboratory data were collected from the electronic medical record (EPD-Vision, Leiden, The Netherlands; Metavision, Itémedical, Tiel, The Netherlands; Hix, Chipsoft, Amsterdam, The Netherlands) and analysed. The EHMRG was calculated retrospectively as previously described [5]. Parameters required to calculate the EMHRG are displayed in Tab. 1. In three patients, oxygen saturation at triage was not registered. Since there were no signs of respiratory distress and supplemental oxygen was not necessary in these patients, oxygen saturation was scored as > 90%. A troponin T level above 51 ng/l was considered elevated. In 23 patients (10%), no troponin T level at triage was available. To avoid underscoring, these patients were considered to have an elevated troponin level. Since metolazone is not registered for clinical use in the Netherlands, thiazide-like diuretics were scored as an alternative.

**Table 1:** Parameters required to calculate the Emergency Heart failure Mortality Risk Grade

Age
Arrival by ambulance
Triage SBP
Triage HR
Triage SpO_2_
Potassium concentration
Creatinine concentration
Troponin level
Active cancer
Metolazone use before ED arrival

*SBP* systolic blood pressure, *HR* heart rate, *SpO*_*2*_ oxygen saturation_,_ *ED* emergency department

Follow-up data were collected from the electronic medical record up to 30 days after a patient visited the emergency department. If care was (partly) continued in another hospital or by the general practitioner, these health care providers were contacted to request the required follow-up data. Mortality was assessed at 7 and 30 days. For patients that were discharged from the emergency department, hospitalisations for decompensated HF at a later time were noted.

### Classification based on EHMRG

Based on their EHMRG, patients were classified into five risk groups, as previously described [4]: very low (EHMRG7 threshold ≤ −49.05), low (EHMRG7 threshold −49.04 to −15.92), intermediate (EHMRG7 threshold −15.91 to 17.97), high (EHMRG7 threshold 17.98 to 56.55), and very high risk (EHMRG7 threshold ≥ 56.56). In line with Lee et al., discharge was considered safe in very low risk and low-risk patients. Hospitalisation was considered necessary in high-risk and very high risk patients [4]. In intermediate-risk patients, we assessed whether there were any criteria for hospitalisation in the coronary care unit or intensive care as mentioned in the ESC guideline (Tab. 2, page 2177) [6]. If any of these criteria were met, hospitalisation was considered necessary. Accordingly, based on their EHMRG, patients were considered safe to discharge or hospitalisation was advised.

**Table 2:** Additional criteria for intermediate-risk group patients regarding admission to the coronary/intensive care unit

Persistent, significant dyspnoea or haemodynamic instability
Acute coronary syndrome
Need for intubation (or already intubated)
Signs/symptoms of hypoperfusion
SpO_2_ < 90% despite supplemental oxygen
Use of accessory muscles for breathing, respiratory rate > 25/min
HR < 40 or > 130 bpm, SBP < 90 mm Hg

*SpO*_*2*_ oxygen saturation, *HR* heart rate, *bpm* beats per minute, *SBP* systolic blood pressure

### Statistical analysis

Continuous variables are expressed as mean ± standard deviation when normally distributed, or as median and interquartile range when not normally distributed. Categorical variables are presented as numbers and percentages. Mortality differences among the different EHMRG risk groups were compared using the Fisher exact test. A McNemar test was performed to evaluate the difference between decision-making based on clinical judgement and the theoretical situation where decision-making would have been based on EHMRG. Analyses were performed with IBM SPSS Statistics for Windows (Version 25.0, Armonk, NY, USA). A *p-*value < 0.05 was considered statistically significant.

## Results

### Study population

A total of 227 unique patients visiting the emergency department because of decompensated HF were included. As shown in Table S1 (Electronic Supplementary Material), mean age was 73 ± 12 years, 157 (69%) were male and 147 (65%) were previously known to have HF. Age, creatinine level and estimated glomerular filtration rate were all EHMRG variables and significantly different among risk groups. N‑terminal pro-B-type natriuretic peptide, which is not an EHMRG variable, was also significantly different among risk groups. Tab. 3 displays the EHMRG variables for the entire study population. At 7‑day follow-up 11 patients (5%) had died, and at 30-day follow-up 25 (11%). Table S2 (Electronic Supplementary Material) shows the use of HF medication prior to the emergency department visit in the 147 patients that had already been diagnosed with HF.

**Table 3:** Emergency Heart failure Mortality Risk Grade variables for the entire study population (*n* = 227)

	Median (IQR) or *n* (%)	
Age, years (SD)	75	(65–85)
Arrival by ambulance	112	(49%)
Triage SBP, mm Hg	140	(120–165)
Triage HR, bpm	74	(85–109)
Triage SpO_2_, %	96	(92–98)
*Potassium concentration*		
< 4.0 mmol/l	48	(21%)
4.0–4.5 mmol/l	76	(36%)
> 4.5 mmol/l	103	(45%)
Creatinine concentration, µmol/l	109	(81–136)
Troponin T level, > 51 ng/l	100	(44%)
Active cancer	11	(5%)
Metolazone use before ED arrival	20	(9%)

*IQR* interquartile range, *SBP* systolic blood pressure, *HR* heart rate, *bpm* beats per minute, *SpO*_*2*_ oxygen saturation, *ED* emergency department

### EHMRG and mortality

**Fig. 1:**
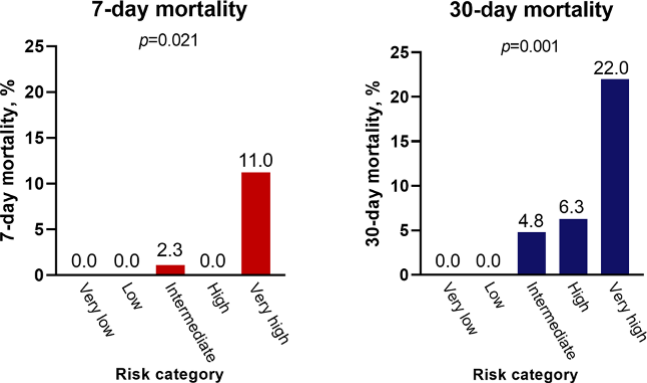
Mortality rates

Based on their EHMRG, 24 patients were classified as very low risk (11%), 22 patients as low risk (10%), 42 patients as intermediate risk (18%), 48 patients as high risk (21%) and 91 patients as very high risk (40%). As shown in Fig. 1, mortality rates were different among the different EHMRG risk groups at 7‑day (*p* = 0.021) and 30-day follow-up (*p* = 0.001). Of note is that in the very low risk and low-risk group, 30-day mortality was 0%. The 30-day mortality rate was 4.8% in the intermediate group, 6.3% in the high-risk group and 22.0% in the very high risk group.

### Hospitalisation versus discharge decision

**Fig. 2:**
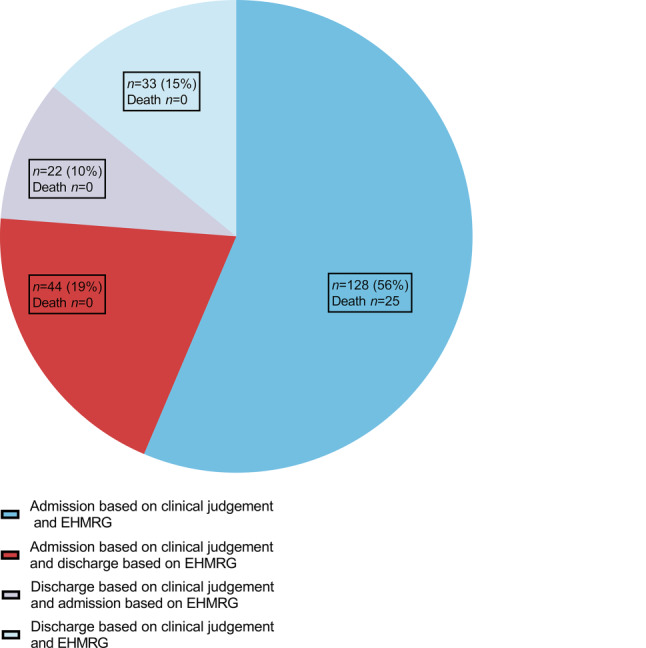
Admitted versus discharged patients

Based on clinical judgement, 172 patients were hospitalised (76%). If decision-making had been based on EHMRG, only 150 patients (66%; *p* < 0.01) would have been hospitalised. Fig. 2 shows that in 161 patients (71%) the outcome of clinical judgement and EHMRG-guided decision-making was similar. In 44 patients (19%) hospitalisation was deemed necessary based on clinical judgement, while the EHMRG indicated that discharge would have been safe. In contrast, discharge was considered safe based on clinical judgement in 22 patients (10%), while the EHMRG indicated that hospitalisation would have been advised. Of note is that in all 25 patients that had died at 30-day follow-up, both clinical judgement and EHMRG indicated that hospitalisation was necessary.

Analysis of the 44 hospitalised patients (mean age 70 ± 10 years, 70% male) that would have been discharged based on their EHMRG revealed that EHMRG classified 12 of these 44 as very low risk and 14 as low risk. Twenty-five of the total of 26 (very) low-risk patients (96%) were treated with intravenous diuretics during hospitalisation. The remaining 18 patients were classified as intermediate risk, but none fulfilled the admission criteria for admission to the coronary care or intensive care unit. The mean hospital stay of these 44 patients was significantly shorter than in the patients admitted on the basis of their EHMRG (7 days vs 8 days ± 0.43; *p* = 0.023).

Analysis of the 22 patients who were discharged based on clinical judgement while the EHMRG advised hospitalisation revealed that the EHMRG classified 12 of these 22 patients as high risk and 10 as very high risk. The reasons for (non-)admission were very different, including the judgement of the treating cardiologist that treatment at home was safe and feasible, the wish of the patient to be treated at home or the decision to choose a palliative policy. None of these 22 patients died within 30 days. Nevertheless, 4 of these 22 patients were hospitalised for decompensated HF within 30 days after discharge from the emergency department. The total number of patients that were (re-)hospitalised for HF within 30 days after the emergency department visit is shown in Table S3 (Electronic Supplementary Material).

## Discussion

The main finding of the current study is that the EHMRG accurately differentiates between high- and low-risk decompensated HF patients visiting emergency departments in the Netherlands, thereby making it a safe and promising tool to guide the hospitalisation versus discharge decision. The current results indicate that routine EHMRG calculation may help to substantially and safely reduce the number of hospital admissions for HF by identifying low-risk patients who can be treated at home.

The yearly number of HF admissions is already high and will, if we proceed as to date, further increase due to ageing of the population [7]. As this poses a substantial burden on health care resources, efforts to reduce potentially avoidable hospitalisations are warranted. The first step in optimising the efficiency of hospitalisations for HF is accurate risk assessment. Although there are numerous risk models for chronic HF patients in the ambulatory setting [8], relatively few prognostic scores have been validated in patients with acute HF. The EHMRG was developed in 2012 based on 12,591 Canadian HF patients visiting the emergency department and aimed to predict mortality within 7 days of presentation [5]. In 2016, Gil et al. applied the EHMRG in 1553 Spanish acute HF patients attending the emergency department [9]. This study, including both palliative and non-palliative patients, showed extrapolation to a cohort with a higher mortality risk, although stratification improved when the score was recalibrated in the Spanish cohort.

In 2019, Lee et al. tested the original EHMRG7 and the new EHMRG30-ST in nearly 2000 Canadian patients in a prospective manner [4]. This cohort included relatively older patients (median 81 years), with 71% having a previous diagnosis of HF. The 7‑day mortality was 2% and 30-day mortality 7%. In this cohort 21% of patients were discharged from the emergency department. Patients assigned to the very low risk and low-risk group had a mortality rate of 0% at 30 days. The findings of the current study are in line with those described by Lee et al., although the current study cohort was younger (mean 73 years), comprised more de novo HF patients and had a higher 30-day mortality. In the current study we assessed EHMRG7, as the EHMRG30-ST calculator is not available yet. Although 30-day mortality was 11% in the entire study population, all patients stratified to the very low risk and low-risk group were alive at 30-day follow-up. With this finding, we externally validated the ability of the EHMRG to predict mortality in a Dutch real-world HF cohort.

Now that we know that the EHMRG can accurately identify low-risk HF patients, the next step in optimising efficiency of hospitalisations for HF is to assure that patients can receive good and safe treatment at home. Apart from mortality risk, previous EHMRG studies already identified other factors that influence the hospitalisation versus discharge decision. These factors include, for instance, self-care ability, availability of social support, multiple active medical conditions requiring treatment simultaneously, patient disease awareness, disease-related behaviour and functional status [4]. However, apart from these factors, which can only be partially influenced, there are others that can be improved. In particular, intravenous diuretic delivery at home has been reported to be safe, feasible and effective in achieving decongestion [10]. If this is accompanied by telemonitoring and home health nurses closely collaborating with the treating cardiologist [11], the patients’ general practitioner and home care organisations, it seems realistic to assume that in the majority of low-risk patients, and even in vulnerable elderly, treatment at home is feasible. If, as suggested by the results of the current study, the number of hospitalisations for HF can thereby be reduced by 10%, this will substantially reduce costs and, last but not least, prevent iatrogenic complications.

### Limitations

There are potential limitations to the present study that should be considered when interpreting the results. Firstly, as the EHMRG was calculated retrospectively, future prospective studies are warranted to confirm the ability of the EHMRG to identify low-risk patients, enable treatment at home and thereby reduce hospital admissions. Secondly, the current study was performed in a single centre. Although both known and de novo HF patients were included, it remains to be investigated whether these data can be extrapolated to other Dutch hospitals. Thirdly, since the novel EHMRG-30ST calculator is not yet available, it remains to be assessed whether this variant of the EHMRG is even more accurate in identifying low-risk patients. Finally, patients with HF secondary to another disease (e.g. acute coronary syndrome or arrhythmias) were not included in the study, since the EHMRG was previously not validated for this group of patients. Moreover, the primary diagnosis may have influenced the decision regarding hospital admission or discharge from the emergency department [4, 5].

## Conclusion

In conclusion, the EHMRG accurately differentiates between high- and low-risk decompensated HF patients visiting the emergency department, making it a safe and promising tool to guide the hospitalisation versus discharge decision in Dutch HF patients.

## Supplementary Information

Table S1. Baseline patient characteristics per EHMRG risk category (*N* = 227)

Table S2. Medication use prior to the emergency department visit in known heart failure patients with preserved, mid-range and reduced ejection fraction (*N* = 147)

Table S3. Total number of patients that were (re)hospitalized for heart failure within 30 days after the emergency department visit per EHMRG risk category (*N* = 227)
