# Supplementary material for: Emergency Heart failure Mortality Risk Grade may help to reduce heart failure admissions
Source: Neth Heart J. 2022 Mar 11;30(9):431–5. doi: 10.1007/s12471-022-01661-3 (PMC9402862; doi:10.1007/s12471-022-01661-3)
Supplement: Supplementary file 1 — Table S1. Baseline patient characteristics per EHMRG risk category (N = 227) [file 12471_2022_1661_MOESM1_ESM.docx]

|  | | | **Table S1. Baseline patient characteristics per EHMRG risk category (N=227)** | | | | |  |  |  |
| --- | --- | --- | --- | --- | --- | --- | --- | --- | --- | --- |
| **EHMRG Risk category** | | | | **Baseline** | **Very Low** | **Low** | **Intermediate** | | **High** | **Very High** |
|  | | | | **N=227** | **N=24** | **N=22** | **N=42** | | **N=48** | **N=91** |
| Age (years)*** | | | | 73±12 | 61±10 | 63±10 | 69±10 | | 76±11 | 77±12 |
| Male | | | | 157(69%) | 16(67%) | 17(77%) | 29(69%) | | 29(60%) | 66(73%) |
| BMI | | | | 27±6 | 29±7 | 29±4 | 29±7 | | 26±5 | 27±6 |
| Previously known with HF | | | | 147(65%) | 13(54%) | 16(72%) | 29(69%) | | 30(63%) | 59(65%) |
| HF aetiology | | | |  |  |  |  | |  |  |
|  | Ischemic | | | 64(28%) | 3(12%) | 7(32%) | 11(26%) | | 14(29%) | 29(32%) |
|  | Non-ischemic | | | 62(27%) | 7(21%) | 7(32%) | 14(33%) | | 14(29%) | 22(24%) |
|  | Unknown at time of ED visit | | | 101(45%) | 16(67%) | 8(36%) | 17(41%) | | 20(42%) | 40(44%) |
| Co-morbidities | | | |  |  |  |  | |  |  |
|  | Hypertension | | | 117(51%) | 11(52%) | 11(50%) | 20(48%) | | 25(52%) | 50(55%) |
|  | Diabetes mellitus | | | 70(30%) | 7(29%) | 6(27%) | 11(26%) | | 10(20%) | 36(40%) |
|  | CVA/TIA | | | 13(6%) | 0(0%) | 3(14%) | 0(0%) | | 3(6%) | 7(7%) |
|  | COPD | | | 22(10%) | 2(8%) | 0(0%) | 4(9%) | | 3(6%) | 13(14%) |
|  | Renal disease^±^ | | | 37(16%) | 2(8%) | 2(9%) | 6(14%) | | 7(15%) | 20(22%) |
|  | Any cancer | | | 27(12%) | 0(0%) | 0(0%) | 2(5%) | | 3(6%) | 6(7%) |
| Cardiac History | | | |  |  |  |  | |  |  |
|  | Previous CAD^¥^ | | | 83(37%) | 7(29%) | 9(41%) | 13(31%) | | 18(38%) | 36(40%) |
|  | Valvular disease | | | 77(34%) | 7(29%) | 10(46%) | 14(33%) | | 16(33%) | 30(33%) |
|  | Atrial fibrillation | | | 107(47%) | 10(42%) | 15(68%) | 16(38%) | | 25(52%) | 41(45%) |
|  | CRT-D or ICD | | | 74(33%) | 2(8%) | 4(18%) | 9(21%) | | 11(23%) | 19(21%) |
| LV function | | | |  |  |  |  | |  |  |
|  | Preserved (EF>50%) | | | 25(11%) | 2(8%) | 2(9%) | 3(7%) | | 5(10%) | 13(14%) |
|  | Mid-range (EF 40-49%) | | | 43(19%) | 5(21%) | 7(32%) | 11(26%) | | 8(17%) | 12(13%) |
|  | Reduced (EF <40%) | | | 79(35%) | 6(25%) | 7(32%) | 15(36%) | | 17(35%) | 34(37%) |
|  | Unknown at time of ED visit | | | 80(25%) | 11(46%) | 6(27%) | 13(31%) | | 18(38%) | 32(35%) |
| Laboratory results | | | |  |  |  |  | |  |  |
|  | | eGFR*** | | 57  (39-74) | 83  (65-90) | 59  (50-76) | 64  (51-81) | | 53  (36-72) | 45  (31-61) |
|  | | Creatinine*** | | 103  (81-136) | 83  (71-92) | 95  (79-111) | 94  (76-119) | | 105  (86-125) | 122  (84-171) |
|  | | NT-pro BNP*** | | 3884  (1880-9287) | 1840  (891-4602) | 2569  (1571-5609) | 2523  (1527-5807) | | 4530  (2703-10028) | 6468  (2480-15144) |
| Medication use | | | |  |  |  |  | |  |  |
|  | Loop diuretic | | | 131(58%) | 11(46%) | 11(50%) | 27(64%) | | 27(56%) | 55(60%) |
|  | Thiazide diuretic* | | | 20(9%) | 0(0%) | 1(5%) | 2(5%) | | 2(4%) | 15(17%) |
|  | ACE inhibitors | | | 88(40%) | 14(58%) | 13(59%) | 13(31%) | | 15(31%) | 33(36%) |
|  | ARB | | | 20(9%) | 2(8%) | 1(5%) | 4(10%) | | 5(10%) | 8(9%) |
|  | Beta-blocker | | | 166(73%) | 19(79%) | 18(81%) | 28(67%) | | 37(77%) | 64(70%) |
|  | MRA | | | 26(12%) | 2(8%) | 3(14%) | 8(19%) | | 5(10%) | 8(9%) |
|  | ARNI | | | 23(10%) | 2(8%) | 1(5%) | 4(10%) | | 4(8%) | 12(13%) |
|  | Digoxin | | | 24(11%) | 2(8%) | 2(9%) | 4(10%) | | 8(17%) | 8(9%) |
|  | Oral anticoagulation | | | 120(53%) | 11(46%) | 16(72%) | 20(48%) | | 27(56%) | 46(51%) |
|  | Statin | | | 126(56%) | 15(63%) | 12(55%) | 22(52%) | | 20(42%) | 57(63%) |

ACE inhibitors= Angiotensin-converting-enzyme inhibitors. ARB=angiotensin receptor blocker. ARNI=Angiotensine neprilysine receptor inhibitor. BMI= body mass index. CAD= coronary artery disease. COPD= indicated chronic obstructive pulmonary disorder. CRT-D= cardiac resynchronisation therapy-defibrillator. CVA= cerebrovascular accident. ED= emergency department. EF=ejection fraction. eGFR= Estimated Glomerular Filtration Rate HF=heart failure. ICD= implantable cardioverter-defibrillator. MRA=Aldosteron antagonist. NT-pro-BNP= N-terminal prohormone of brain natriuretic peptide. TIA= transient ischemic attack.
*** P < 0.001 between the different risk categories
± defined as chronic kidney disease in medical history
¥ defined as percutaneous coronary intervention (PCI) or coronary artery bypass graft (CABG
* p < 0.05 between the different risk categories
