# Supplementary material for: Emergency Heart failure Mortality Risk Grade may help to reduce heart failure admissions
Source: Neth Heart J. 2022 Mar 11;30(9):431–5. doi: 10.1007/s12471-022-01661-3 (PMC9402862; doi:10.1007/s12471-022-01661-3)
Supplement: Supplementary file 2 — Table S2. Medication use prior to the emergency department visit in known heart failure patients with preserved, mid-range and reduced ejection fraction (N = 147) [file 12471_2022_1661_MOESM2_ESM.docx]

| **Table S2. Medication use prior to the emergency department visit in known heart failure patients with preserved, mid-range and reduced ejection fraction (N=147)** | | | |
| --- | --- | --- | --- |
|  | **Preserved (EF>50%)** | **Mid-range (EF 40-49%)** | **Reduced (EF <40%)** |
|  | **N=25** | **N=43** | **N=79** |
| Loop diuretic | 14(56%) | 30(70%) | 67(85%) |
| Thiazide diuretic | 4(16%) | 1(2%) | 7(9%) |
| ACE inhibitors | 11(44%) | 17(40%) | 38(49%) |
| ARB | 6(24%) | 4(9%) | 8(10%) |
| Beta-blocker | 18(72%) | 33(77%) | 68(86%) |
| MRA *** | 0(0%) | 1(2%) | 20(25%) |
| ARNI | 1(4%) | 7(16%) | 13(17%) |
|  |  |  |  |

ACE inhibitors= Angiotensin-converting-enzyme inhibitors. ARB=angiotensin receptor blocker. ARNI=Angiotensine neprilysine receptor inhibitor. ED= emergency department. EF=ejection fraction. MRA=Aldosteron antagonist.
*** P < 0.001
