# Supplementary material for: Emergency Heart failure Mortality Risk Grade may help to reduce heart failure admissions
Source: Neth Heart J. 2022 Mar 11;30(9):431–5. doi: 10.1007/s12471-022-01661-3 (PMC9402862; doi:10.1007/s12471-022-01661-3)
Supplement: Supplementary file 3 — Table S3. Total number of patients that were (re)hospitalized for heart failure within 30 days after the emergency department visit per EHMRG risk category (N = 227) [file 12471_2022_1661_MOESM3_ESM.docx]

| **Table S3. Total number of patients that were (re)hospitalized for heart failure within 30 days after the emergency department visit per EHMRG risk category (N=227)** | | | | | |
| --- | --- | --- | --- | --- | --- |
| **EHMRG Risk category** | **Very Low** | **Low** | **Intermediate** | **High** | **Very High** |
|  | **N=24** | **N=22** | **N=42** | **N=48** | **N=91** |
| (Re)admission | 2 (8%) | 2(9%) | 3(7%) | 5(10%) | 7(7%) |
|  |  |  |  |  |  |
